# Signaling by epithelial members of the CEACAM family – mucosal docking sites for pathogenic bacteria

**DOI:** 10.1186/1478-811X-12-27

**Published:** 2014-04-15

**Authors:** Arnaud Kengmo Tchoupa, Tamara Schuhmacher, Christof R Hauck

**Affiliations:** 1 Lehrstuhl für Zellbiologie, Universität Konstanz, 78457 Konstanz, Germany; 2 Konstanz Research School Chemical Biology, Universität Konstanz, 78457 Konstanz, Germany

**Keywords:** Signal transduction, GPI anchor, Membrane microdomains, Endocytosis, CEACAM

## Abstract

Carcinoembryonic antigen-related cell adhesion molecules (CEACAMs) comprise a group of immunoglobulin-related vertebrate glycoproteins. Several family members, including CEACAM1, CEA, and CEACAM6, are found on epithelial tissues throughout the human body. As they modulate diverse cellular functions, their signaling capacity is in the focus of current research. In this review we will summarize the knowledge about common signaling processes initiated by epithelial CEACAMs and suggest a model of signal transduction by CEACAM family members lacking significant cytoplasmic domains. As pathogenic and non-pathogenic bacteria exploit these receptors during mucosal colonization, we try to highlight the connection between CEACAMs, microbes, and cellular responses. Special emphasis in this context is placed on the functional interplay between CEACAMs and integrins that influences matrix adhesion of epithelial cells. The cooperation between these two receptor families provides an intriguing example of the fine tuning of cellular responses and their manipulation by specialized microorganisms.

## Introduction

**Figure 1:**
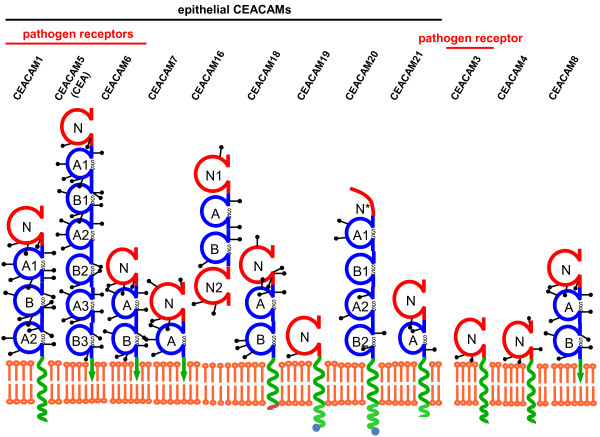
**The human CEACAM family.** Schematic depiction of the twelve members of the human carcinoembryonic antigen-related cell adhesion molecules. The red spheres indicate Ig_V_-like domains, the blue spheres indicate Ig_C2_-like domains, which are stabilized by disulfide bonds (S-S). The green spirals indicate transmembrane helices. GPI-anchors are depicted in the form of a green arrow ending in the lipid bilayer. CEACAM20 encodes only a partial Ig_V_-like domain (N*). Graph modified from http://www.carcinoembryonic-antigen.de/.

The *c*arcino*e*mbryonic *a*ntigen-related *c*ell *a*dhesion *m*olecules (CEACAMs), a subgroup of the CEA family of immunoglobulin-related proteins, are encoded in the human genome by 12 genes [1,2] (Figure 1). All 12 expressed *CEACAM* genes and a number of derived pseudogenes cluster on chromosome 19q13 [3,4]. CEACAMs show distinct expression patterns on different cell types [1,5]. Whereas particular CEACAMs are only expressed in certain epithelial or myeloid cells, others are found in various tissues [6]. Some family members play a precise functional role in particular events such as hearing in the inner ear (CEACAM16) or phagocytosis of specific bacterial pathogens (CEACAM3) [7,8]. However, most CEACAMs can be seen as modulators of general cellular processes such as cell adhesion, differentiation, proliferation, and survival. To fulfill such diverse functions, CEACAMs have to intersect with other cellular receptors and to transmit signals into the cell. Indeed, signal transduction mediated by distinct CEACAM family members, which encompass a cytoplasmic domain, such as CEACAM3 and a splice variant of CEACAM1 with long cytoplasmic domain, has been studied in great detail [6,9]. Given the fact that several CEACAMs are GPI-anchored proteins or that they sustain functionality in the absence of a cytoplasmic domain, the mechanistic details of signal transduction processes initiated by these CEACAM family members are still widely unresolved. Interestingly, CEACAMs are utilized by bacterial pathogens as host receptors on epithelial cells. Similar to physiological stimulation of CEACAMs, bacteria-initiated clustering of CEACAMs can induce robust cellular responses including activation of certain kinases, stimulation of small G proteins, cytoskeletal rearrangements, induction of novel gene expression events, enhanced cell adhesion, and receptor endocytosis. It has become clear that CEACAM-binding bacterial pathogens exploit the signaling capacity of these immunoglobulin superfamily receptors to enhance their chances of colonizing the mucosal surface. As CEACAM family members without significant cytoplasmic domains dominate on several epithelial surfaces such as breast, liver, or prostate [10], we will use this review, to summarize the current knowledge about the signaling function of these epithelial CEACAMs. By highlighting recent advances in the understanding of bacteria-induced CEACAM-mediated processes, we provide a framework for further dissecting the molecular signaling connections emanating from epithelial members of this family.

## Physiological roles of epithelial CEACAMs

Since the discovery of carcinoembryonic antigen (CEA) some 50 years ago [11], and the subsequent appreciation of a family of CEA-related cell adhesion molecules [12] (Figure 1), numerous physiological and pathological processes have been associated with these mammalian membrane glycoproteins. Historically, cancer is one of the disease states linked to aberrant CEACAM function and the role of epithelial CEACAMs in tumour progression and metastasis has been summarized in an excellent review recently [13]. In particular, human CEACAM1, CEA, and CEACAM6, which can be found on various epithelial cell types and derived carcinomas, are thought to shape the interaction between tumour cells and their stromal counterparts as well as immune cells. Apart from their potential utilization as clinical biomarkers and promising therapeutic targets in melanoma, lung, colorectal, and pancreatic cancers, these epithelial CEACAMs are also implicated in morphogenesis [14,15], angiogenesis [16,17], cell proliferation [18], cell motility [19,20], apoptosis [21], regulation of cell matrix attachment [22,23], as well as epithelial cell-cell interaction and cell polarisation [24,25]. Clearly, forward and reverse genetic approaches in animal models have suggested that CEACAMs are not essential for all these processes. For example, mice lacking CEACAM1 are viable and fertile and do not show gross morphological alterations [26]. Furthermore, heterologous expression of human CEACAM1 in the mouse or expression of additional human epithelial CEACAMs, which are not encoded in the murine genome (such as CEA and CEACAM6), does not result in perturbation of tissue architecture or normal tissue homeostasis [27-29]. Therefore, epithelial CEACAMs seem to contribute to the fine-tuning of cellular behaviour and their contribution might become critical during stressful conditions, such as tissue damage and repair, which are not readily obvious in laboratory kept animals.

Most studies of CEACAM-initiated signal transduction have focussed on CEACAM1 in immune cells and transformed epithelial cells (nicely summarized in [6,13]). Investigations into CEACAM1 structure and function have also profited from the fact that this family member is expressed in different cell types and that CEACAM1 orthologs exist in other mammalian species [30]. Due to differential splicing, human CEACAM1 occurs in 11 isoforms with the number of extracellular Ig domains ranging from one to four (see the CEA homepage at http://www.carcinoembryonic-antigen.de/index.html; [31]). The major isoforms in human cells are CEACAM1-4 and CEACAM1-3, which possess an extracellular amino-terminal Ig_V_-like domain, followed by three (A1, B, A2) or two (A1, B) Ig_C2_-like domains, respectively. Similarly, in other epithelial CEACAMs, such as CEA or CEACAM6, up to six extracellular Ig_C2_-like domains follow the amino-terminal Ig_V_-like domain (Figure 1). Accordingly, engagement of the extracellular domains of epithelial CEACAMs serves as the primary stimulus for CEACAM-mediated transmembrane signaling. Under physiologic conditions, homophilic interactions between CEACAMs on opposing cells are thought to be the major trigger of CEACAM-initiated signaling processes, although CEACAMs can also engage in heterophilic interactions, e.g. with selectins [32].

## Role of CEACAM extracellular domains in mediating cis- and trans-oligomerization

*Trans*-oligomerization resulting from homophilic interactions between the amino-terminal Ig_V_-like domains of CEACAMs on neighbouring epithelial cells is the basis of CEACAM-mediated cell-cell adhesion [33-36]. However, it has become clear that this homophilic type of *trans*-oligomerization is further supported by the presence of Ig_C2_-like domains [33,37]. In a tissue context, these additional extracellular Ig domains might allow these receptors to extend farther from the membrane surface to facilitate binding, but they might also be directly involved in homophilic *trans*-interactions [33,38]. Moreover, recent electron tomography studies of soluble and membrane-attached CEACAM1 ectodomains have not only confirmed the critical role of the Ig_V_-like amino-terminal domain for *trans*-oligomerisation, but also pointed to additional *cis*-interactions in the extracellular part of CEACAM1 [39]. Indeed, the extracelluar chain of Ig domains in CEACAM1 appears to be rather flexible, but can be stabilized by *cis*-interactions between either Ig_V_-like domains or Ig_C2_-like domains of parallel CEACAM1 molecules in the same membrane plane [39]. As a consequence, CEACAMs might occur in different oligomerization states, partially dictated by the occurrence of *trans*- or *cis*-interactions between their extracellular domains. At least in the case of CEACAM1, these different oligomerization states clearly have an influence on its signaling function [40]. In one of the following sections, it will become clear that the issue of CEACAM1 oligomerization is even more complex, as the transmembrane domain of this receptor also sustains *cis*-interactions, presumably depending on the lipid context.

## Signaling by epithelial CEACAMs

As transmembrane signaling requires a connection to the cytosol, the transmembrane domain containing CEACAM1 has been the focus of a multitude of studies [6,13]. Indeed, CEACAM1 harbors a cytoplasmic domain, which can either be long (L; 71 amino acids in humans) or short (S; 10 amino acids). The “L” isoforms encompass a functional immunoreceptor tyrosine-based inhibitory motif (ITIM) and both CEACAM1-L and CEACAM1-S isoforms are often co-expressed in the same cell, with expression ratios varying between different cell types and between different cellular states [18,41]. In many cases, expression of the short isoform interferes with CEACAM1-L generated signals [40,42]. Therefore, the signal transduction role of CEACAM1 has been mostly attributed to the CEACAM1-L isoform and its cytoplasmic domain. Indeed, CEACAM1-L can interact with cytoplasmic protein tyrosine kinases and protein tyrosine phosphatases, as well as with calmodulin, β-catenin, actin, filamin, shc, and tropomyosin (for review see [13]). Only few of these interactions are sustained by the short cytoplasmic domain of CEACAM1-4S. However, investigations of transformed mammary epithelial cells (MCF7 cells) grown in a 3D-matrigel environment have suggested that CEACAM1-4S can induce lumen formation in these carcinoma cells resulting in acinar-like structures [14]. In follow up studies, the effect of CEACAM1-4S was pinpointed to binding interactions of the short cytoplasmic domain. In particular, in CEACAM1-4S the membrane-proximal phenylalanine F454 or lysine K456 residues (-H**F**G**K**TGSSGPLQ), respectively, interact with cytoskeletal components and T457 (-HFGK**T**GSSGPLQ) is phosphorylated [43]. Furthermore, MCF7 cells injected together with human fibroblasts in the fat pad of mice show a more normal phenotype (with lumen formation), when CEACAM1 is stably expressed in these cells [44]. In this situation, both CEACAM1-4S and CEACAM1-4L are able to induce lumen formation and gland development in the xenograft [45]. Therefore, despite major differences in their cytoplasmic sequences and their distinct profiles of protein-protein interactions, both CEACAM1-4L as well as CEACAM1-4S appear to modulate the growth behaviour of epithelial cells in a similar manner. These findings imply that they can transmit at least some overlapping signals into the cells. Indeed, phosphorylation of the membrane proximal threonine residue (T457), present in the cytoplasmic domains of CEACAM1-4S and CEACAM1-4L, by calmodulin kinase IID (CaMKIID) is the critical event required for CEACAM1-driven lumen formation in transformed breast epithelial cells [46].

A similar contribution of CEACAM1 to morphogenesis has now been reported in 3D cultures of prostate cells [47]. The primary human prostate cells formed organoids with a lumen and small tubular outgrowth, which was inhibited, when anti-CEACAM1 antibodies were added to the cultures or when CEACAM1 expression was reduced by about 50% with antisense oligonucleotides [47]. As these cells express both CEACAM1 isoforms, with either short or long cytoplasmic domain, it is unclear if one or both proteins are responsible for the phenotype. Prostate epithelial cells express an additional member of the CEACAM family, CEACAM20, which is found together with CEACAM1 on the luminal surface of normal prostate glands. Again, antisense oligonucleotides against CEACAM20 reduced tubule outgrowth [47]. Clearly, CEACAM20 has a cytoplasmic domain sequence distinct from CEACAM1. Even more striking, CEACAM20 lacks a complete Ig_V_-like amino-terminal domain, which is instrumental in CEACAM1 for homophilic interactions between CEACAM1 on neighbouring cells. Together, these recent insights point to functional commonalities between epithelial CEACAM family members, which show striking sequence divergence in their amino-terminal Ig_V_-like domain or their cytoplasmic sequences.

One important implication arising from these results is the realization that signaling by epithelial CEACAMs could involve parts of these receptors other than the cytoplasmic domain or the amino-terminal Ig_V_-like domain, such as the transmembrane or additional extracellular domains. Indeed, recent experiments employing either carcinoma cell lines or using bacterial pathogens as CEACAM ligands have pointed into this unexpected direction.

## CEACAM1 *cis*-oligomerization sustained by the transmembrane domain

**Figure 2:**
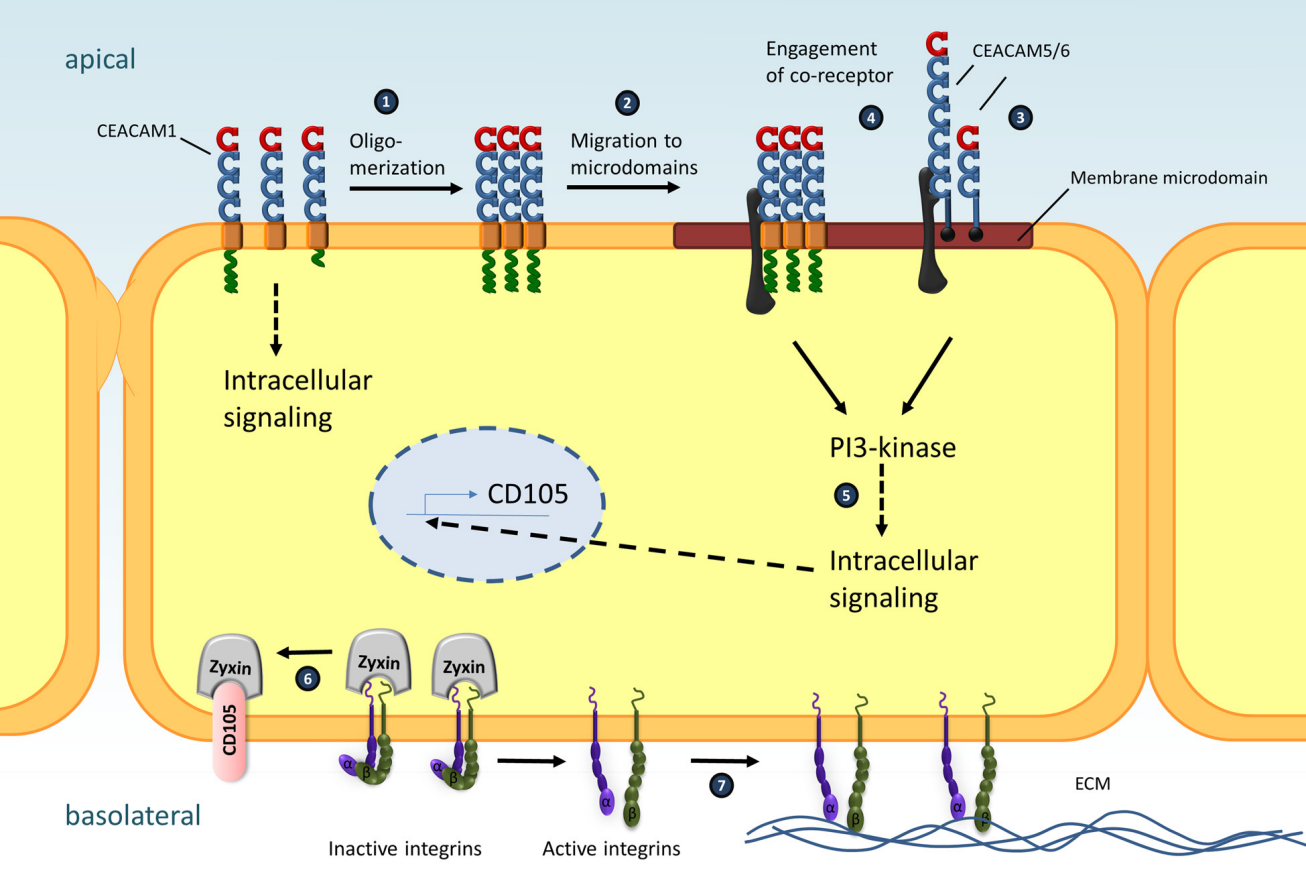
**Signaling initiated by epithelial CEACAMs.** Schematic summary of recent findings with regard to CEACAM-initiated signaling events in epithelial cells. Upon ligand binding, CEACAM1 forms oligomers supported by cis-interactions between the extracellular and the transmembrane domains (1) and is recruited to membrane microdomains (2). GPI-anchored epithelial CEACAMs, such as CEA or CEACAM6, constitutively localize to membrane microdomains (3). In membrane microdomains, epithelial CEACAMs connect to putative co-receptor(s) (black) via extracellular Ig_C2_-like domains (4). Intracellular signaling triggered by epithelial CEACAMs either directly or indirectly via co-receptor(s) leads to phosphatidylinositol-3’-kinase dependent signaling processes connected to receptor-mediated endocytosis (5). Furthermore, stimulation of epithelial CEACAMs triggers novel gene expression events, e.g. *de novo* expression of CD105, which extracts zyxin from basal integrin-rich focal adhesion sites (6), resulting in increased integrin activity and enhanced binding to the basal extracellular matrix (ECM) (7).

A long standing observation in the field is the reduced expression of CEACAM1 that accompanies transformation of epithelial cells from different tissues [13], including the transition from hepatocytes to hepatoma cells. It is therefore not surprising, that re-expression of CEACAM1-4L in rat hepatocellular carcinoma cells results in growth suppression in vitro and reduced tumour formation in vivo [48]. In contrast, expression of CEACAM1-4S in an anchorage-dependent hepatocellular carcinoma cell line promoted robust growth of the cells in soft-agar, suggesting that CEACAM1-4S-initiated signaling rendered the cells anchorage-independent [49]. Strikingly, this effect could be abolished by mutations in the transmembrane domain. In particular, point mutations disrupting a membrane-integral GxxxG motif resulted in the loss of the anchorage-independent growth promoting properties of CEACAM1-4S. As GxxxG motifs in α-helical domains are known to support helix-helix interactions, it was proposed that such mutations might disrupt *cis*-dimer formation of CEACAM1. Recent biochemical approaches based on chemical crosslinking support the idea that CEACAM1 oligomerizes laterally via the transmembrane domain to sustain downstream function [50]. Together, these results indicate that the transmembrane domain of CEACAM1 promotes clustering and oligomerization of the receptor as a pre-requisite for signaling into the cell (Figure 2).

## CEACAM-binding bacteria reveal the lipid raft association of their receptors

Further insight into CEACAM signaling connections has been gained by the use of bacterial pathogens as selective and multivalent stimuli of these receptors. Over the last two decades, diverse CEACAM-binding pathogens including pathogenic *Escherichia coli* strains, *Neisseria gonorrhoeae*, *Neisseria meningitidis*, *Haemophilus influenzae*, and *Moraxella catarrhalis*, have been found to bind to CEACAM1 or other epithelial CEACAMs such as CEA and CEACAM6 [51-56]. In an intriguing example of convergent evolution, these bacteria employ structurally distinct adhesive surface proteins (adhesins) to connect to the same group of human receptors (Table 1). As CEACAM1, CEA, and CEACAM6 are exposed on the apical membrane of mucosal cells, they provide an accessible handle for incoming bacteria (for review see [2]). Indeed, all CEACAM-binding pathogenic bacteria characterized so far exploit the human mucosa as a platform for colonization, multiplication and further spread [57]. In addition to mere binding to host cells, CEACAM engagement triggers endocytosis of the bacteria into epithelial cells and transcytosis of microorganisms through intact epithelial layers [53,58,59]. In this respect, it has been reported before that GPI-anchored CEA and CEACAM6 as well as CEACAM1 initiate a characteristic uptake pathway that is distinct from phagocytosis mediated by the granulocyte receptor CEACAM3 [60,61]. Due to its exceptional phagocytosis promoting properties, CEACAM3-initiated signaling has been studied in great detail (for review see [9]). In contrast to epithelial CEACAMs, CEACAM3-initiated uptake of bacteria critically relies on a cytoplasmic sequence motif and involves extensive actin cytoskeleton rearrangements orchestrated by the small GTPase Rac and its effector protein WAVE2 [8,62]. Importantly, CEACAM3-mediated phagocytosis is independent of sphingolipid- and cholesterol-rich membrane microdomains, as cholesterol chelators do not interfere with this process [61,63]. This is strikingly different for epithelial CEACAMs, where internalization of bacteria is sensitive against cholesterol depletion [61,64]. Therefore, in addition to receptor dimerization and oligomerization, it appears that signaling initiated by epithelial CEACAMs also requires the proper lipid environment in the membrane. For GPI-linked CEA and CEACAM6 it is known for some time that these glycoproteins localize to detergent-resistant membrane fractions [65]. In this regard, the GPI anchor of CEA is sufficient to localize proteins to membrane microdomains [66]. Transmembrane CEACAM1 has also been found in detergent-resistant membrane microdomains in epithelial and endothelial cells [20,67]. In contrast to GPI-anchored CEACAMs, which constitutively localize to the detergent-resistant membrane fraction, CEACAM1 is only found in membrane microdomains upon receptor clustering [67]. This suggests an additional layer of regulation, which drives this receptor into specific membrane regions upon receptor engagement. As mutations in the transmembrane, but not the cytoplasmic domain of CEACAM1 affect localization in detergent-resistant membrane fractions [67], it is tempting to speculate that the receptor oligomerization function of the CEACAM1 transmembrane domain directs this receptor into membrane microdomains (Figure 2). Together, epithelial CEACAMs require a specific lipid environment in the plasma membrane for proper function, where GPI-anchored CEACAMs constitutively localize and where CEACAM1 can be recruited to upon receptor oligomerization.

**Table 1:** CEACAM-binding bacteria and their adhesive proteins

**Bacterial species**	**Primary target tissue**	**Adhesin**	**CEACAM specificity**				**References**
			**1**	**3**	**5**	**6**	
**Adherent-invasive *Escherichia coli *** **(AIEC)**	Digestive tract	FimH	-	NC	-	+	[68]
**Diffusely adhering *Escherichia coli *** **(DAEC)**	Digestive tract Urogenital tract	Afa/Dr-I	+	-	+	+	[69]
***Haemophilus influenzae***	Nasopharynx	OmpP5	+	NC	+	NC	[55,70]
***Moraxella catarrhalis***	Nasopharynx	UspA1	+	+	+	NC	[56,71]
***Neisseria gonorrhoeae***	Urogenital tract	Opa_CEA_	+	+	+	+	[54,72]
***Neisseria meningitidis***	Nasopharynx	Opa_CEA_	+	+	+	+	[53]
**commensal *Neisseria***	Nasopharynx	Opa_CEA_	+	NC	NC	NC	[73]
***Salmonella sp***	Digestive tract	Uncharacterized fimbrial adhesin	+	NC	+	+	[51]

NC: not yet characterized.

## CEACAM1 signaling initiated by the Ig_C2_-like extracellular domains

Though the localization in membrane microdomains is shared by epithelial CEACAMs this does not provide a direct explanation for their signaling capacity. Again, CEACAM-mediated internalization of bacterial pathogens provided novel insight into how epithelial CEACAMs might be mechanistically connected to intracellular signaling pathways. In numerous endocytic processes, phosphatidylinositol phosphates (PIPs) play an important role [74,75]. Therefore, the observation that CEACAM3–mediated internalization is not blocked by wortmannin, an inhibitor of phosphatidylinositol-3’ kinase (PI3K), was particularly striking [76]. This surprising finding with regard to CEACAM3 prompted an investigation of PI3K and PIPs in bacterial internalization via epithelial CEACAMs. Interestingly, in CEACAM1-expressing cells, a strong accumulation of phosphatidylinositol 3’,4’,5’-trisphosphate (PI3,4,5P) was observed around bacterial uptake sites [77]. Furthermore, overexpression of class I PI3K increased bacterial uptake, whereas wortmannin blocked CEACAM1-, CEA-, and CEACAM6-mediated internalisation. Expression of the 5’-phosphate-directed PIP phosphatase SHIP (SH2 domain-containing inositol phosphatase), which dephosphorylates PI3,4,5P, reduces CEACAM1-mediated internalization. Intriguingly, PI3K-dependent endocytosis via CEACAM1 was not linked to cytoplasmic determinants of the receptor, but rather required the extracellular Ig_C2_-like domains of CEACAM1 [77]. Accordingly, expression of CEACAM1 mutants lacking either one or all Ig_C2_-like domains resulted in lower numbers of endocytosed bacteria in comparison to wildtype CEACAM1 despite a similar binding of the microorganisms to the truncated receptor. It is interesting to note, that inhibition of PI3K by wortmannin did not interfere with the re-location of CEACAM1 to membrane microdomains, suggesting that PI3K signaling is downstream of receptor oligomerization and membrane microdomain association of the receptor. A plausible explanation would be that the Ig_C2_ domains of CEACAM1 connect bacteria-bound CEACAM1, presumably via the extracellular part of a membrane-microdomain located receptor, with PI3K signaling inside cells (Figure 2).

It is interesting to note that the IgC2 domains of CEACAM1 orthologs from human, cattle, mouse and rat show a higher degree of sequence conservation than the amino-terminal Ig_V_-like domain [30,78]. The lower sequence conservation in the amino-terminal Ig_V_-like domain compared to the Ig_C2_-like domains has always been interpreted as a sign of positive selection for sequence variation in the amino-terminal domain. However, together with the loss of function upon deletion of the Ig_C2_ domains, the relative conservation of the Ig_C2_ domains of epithelial CEACAMs may reflect conserved functions and therefore evolutionary constraints on this region. Importantly, whereas all isoforms of CEACAM1, CEA, and CEACAM6 encompass at least one Ig_C2_-like extracellular domain, CEACAM3 lacks such an extracellular domain. The absence of an Ig_C2_-like extracellular domain in CEACAM3 correlates well with the mechanistically distinct endocytosis mediated by CEACAM3 in comparison to epithelial CEACAMs. Altogether, it is very tempting to speculate that engagement of epithelial CEACAMs will promote the association of their extracelluar Ig_C2_ domain(s) with not yet identified co-receptor(s), which in turn transmit the PI3K activating signal into the cell (Figure 2). This model would also explain why CEACAMs with differences in the amino-terminal and the cytoplasmic domain (such as CEACAM1 and CEACAM20) can promote similar cellular responses as discussed above for prostate morphogenesis. Such a common co-receptor for multiple CEACAMs might also be located in membrane microdomains, where CEACAM1 re-locates upon oligomerization and where GPI-anchored CEACAMs constitutively localize. The identification of this putative co-receptor might be the turning point in the quest to completely understand the fascinating physiology of epithelial CEACAMs.

## CEACAM cooperation with integrins and other membrane receptors

Several cellular receptors have already been proposed to act as co-receptors for CEA or to co-operate with epithelial CEACAMs [79-81]. For example, in lung epithelial cells, CEACAM1 has been shown to co-immunoprecipitate with Toll-like receptor 2 (TLR2) and bacterial engagement of CEACAM1 has been suggested to interfere with TLR2-induced pro-inflammatory responses [80]. However, as the *Moraxella catarrhalis* strain O35E employed in these studies does not bind to any CEACAM [71], it is unclear, how CEACAM-initiated responses are triggered in this context.

In several studies, it has been observed that CEACAM stimulation has a positive effect on cell-matrix adhesion of epithelial cells as well as on integrin-mediated cell-cell adhesion in leukocytes [20,22,82]. In the case of CEACAM1, a phosphorylation-dependent interaction with integrin β3 has been reported [83] and CEACAM1 colocalizes with integrin β1 in MCF7 cells grown in Matrigel [84] suggesting that CEACAMs functionally interact with integrins. Since ligand-bound integrins locally organize membrane microdomains, they could constitute a co-receptor for epithelial CEACAMs [85,86]. Indeed, the observed functional co-operation was suggested to result from co-clustering of GPI-linked CEACAMs together with integrins in these membrane areas [87]. A co-operation between CEACAMs and integrins would nicely explain the modulation of cellular functions such as cell adhesion and cell survival in the absence of matrix-attachment [88,89]. However, biochemical evidence for a close physical interaction between CEA or CEACAM6 and integrins is lacking. Furthermore, CEACAMs localize to lateral cell-cell contacts or the apical membrane compartment in polarized cells, whereas ligand-bound integrins cluster at basal cell-matrix adhesion sites. The seeming contradiction between functional cooperation and distinct subcellular localization of epithelial CEACAMs and integrins has been nicely resolved. Using CEACAM-binding bacteria as a naturally occurring, highly selective and multivalent ligand for CEACAM1, CEA, and CEACAM6, an unbiased gene-expression analysis revealed a number of genes, which are specifically induced following CEACAM stimulation in epithelial cells [22]. Further analysis showed that upregulation of a member of the TGF-β1 receptor family, termed endoglin or CD105, is observed upon stimulation of GPI-anchored CEACAMs or stimulation of a CEACAM1 mutant lacking the complete cytoplasmic domain [22]. In all these cases, CEACAM engagement by bacteria results in an elevated CD105 mRNA level, which is observed within 1–3 hours after bacterial infection [22]. In a similar time frame, the infected epithelial cells display enhanced integrin-mediated adhesion to the extracellular matrix and CD105 expression is necessary and sufficient for this phenotype [22]. CD105 expression in turn does not alter the amount of integrins on the cells, but initiates the redistribution of the focal adhesion protein zyxin. Indeed, zyxin binds with high affinity to the cytoplasmic domain of CD105, and disappears from integrin-rich focal adhesion sites as soon as CD105 is expressed in epithelial cells [90] (Figure 2). Due to the lack of zyxin at focal adhesions, integrin activity, and therefore extracellular matrix (ECM) binding of the infected cells, increases over the course of several hours following contact with CEACAM-binding bacteria. Increased integrin activity and strengthened ECM-binding is also observed in zyxin-deficient or CD105-overexpressing cells, suggesting that CEACAM-binding bacteria exploit physiological regulators of cell adhesion to indirectly manipulate integrin activity [90] (Figure 2). This functional interplay between CEACAM stimulation, CD105 expression and its effect on focal adhesion site composition provide a plausible scenario, how CEACAMs can modulate integrin-mediated cell adhesion even without directly associating with integrins. However, it should be noted that several CEACAM-binding bacteria also possess surface adhesins, which associate with extracellular matrix (ECM) proteins of their host, such as fibronectin or vitronectin [56,57,71]. In this manner, ECM protein binding could allow such bacteria to simultaneously engage integrins and CEACAMs once the integrity of the epithelial barrier and the spatial separation of CEACAMs and integrins might be compromised. If such a potential co-stimulation of integrins and CEACAMs by pathogenic microbes has consequences for the outcome of bacteria-host interaction has not been explored so far.

Nevertheless, already the indirect connection between CEACAMs and integrins must be advantageous for bacteria trying to get a foothold on the mucosal surface, given the fact that so many unrelated microbes target CEACAMs (Table 1). Indeed, this functional connection allows bacteria to engage receptors on the apical side of the epithelium, while ultimately impacting on the activity of integrins, which are located on the basolateral side of polarized epithelial cells. In the case of CEACAM-binding *Neisseria gonorrhoeae*, which infects the urogenital tract, it has been observed that the enhanced matrix binding of the infected epithelial cells strongly reduces the exfoliation of the superficial mucosal cell layer [90]. Suppressing CD105 upregulation or inhibiting the zyxin-CD105 interaction in the urogenital tract of CEA-transgenic mice allows exfoliation to proceed despite the presence of CEACAM-binding bacteria, providing experimental proof that CEACAM engagement is instrumental for successful colonization of the mucosal surface [90]. Further examples have emerged, which demonstrate that colonization of the nasopharyngeal mucosa by *Neisseria meningitidis* or *Moraxella catarrhalis* profits from the presence of epithelial CEACAMs [91,92]. In the case of *N. meningitidis*, bacteria are not detected in wildtype mice three days after inoculation, whereas the same bacterial strain is present for up to a week in the nasopharynx of CEACAM1-transgenic mice [91]. It is currently unclear, if suppression of epithelial exfoliation, CEACAM-integrin cooperation, or other forms of CEACAM-initiated cellular responses are involved in nasopharyngeal colonization. However, these examples again demonstrate that epithelial CEACAMs, either with or without cytoplasmic domain, can orchestrate signaling events in epithelial cells. Furthermore, they also showcase that much of CEACAM functionality can be learned by using CEACAM-binding bacteria, such as *N. gonorrhoea*e, as selective and potent stimuli.

## Conclusions

Over the last decade, CEACAMs have emerged as important modulators of signaling events in leukocytes, endothelial, and epithelial cells. The simultaneous expression of multiple CEACAM family members by most human epithelial tissues, including GPI-anchored and transmembrane forms in different splice variants, has hampered progress in deciphering the molecular signaling connections initiated by CEACAM-mediated cell-cell interactions. To understand the contribution of CEACAMs in these processes, well characterized antibodies have been employed to interfere with CEACAM-CEACAM interactions, but due to sterical hindrance such approaches might also block a number of other cell-cell interactions. The use of CEACAM-binding bacteria as multivalent, high affinity ligands for a number of epithelial CEACAMs has provided an additional opportunity to selectively trigger CEACAM signaling in vitro and in vivo. These natural probes allow the visualization of local CEACAM-initiated signaling complexes as well as signaling intermediates and, therefore, have provided novel insight. Combining these different approaches will further help to refine our understanding of epithelial CEACAM physiology and of the involved molecular and cellular processes.

## Abbreviations

CEA: Carcinoembryonic antigen; CEACAM: CEA-related cell adhesion molecule; Ig: Immunoglobulin; ITIM: Immunoreceptor tyrosine-based inhibitory motif; PI3K: Phosphatidylinositol-3’ kinase; PTK: Protein tyrosine kinase; TLR2: Toll-like receptor 2.

## Competing interests

The authors declare that they have no competing interests.

## Authors’ contributions

AKT and CRH wrote the manuscript. AKT, TS, and CRH revised the manuscript. All authors read and approved the final manuscript.
